# 554 Preventing accidental central line removal: Early success through a novel securement device

**DOI:** 10.1093/jbcr/irac012.182

**Published:** 2022-03-23

**Authors:** Aemilio W Ha, Mini Thomas

**Affiliations:** UCI Health, Orange, California; UCI Health, Orange, California

## Abstract

**Introduction:**

Central Venous Catheter (CVC) placement and maintenance can be difficult due to non-intact skin and possible surrounding weeping wounds. In addition, routinely used products require intact skin for the adherence of the central line dressing along with sutures or securement devices. With the increased number of line dislodgements, the Burn Intensive Care Unit (BICU) searched for alternative means of line securement. A subcutaneously anchored sutureless system (SASS) was found that could secure the line and allow for improved cleaning around the insertion site. The SASS is a device made of metal which resides in subcutaneous portion of the skin and anchors the CVC while allowing lifting of the catheter to clean underneath.

**Methods:**

After identification of the SASS product, a trial was implemented. Nurses (RN) and providers were trained virtually by the product representative. Nursing champions were selected to be a liaison between the product representative and the staff. Patients included burn patients or Steven Johnsons (SJS) patients, with large open total body surface area, who had a CVC placed peripherally or centrally between September 2020 to June 2021. A survey was conducted upon insertion of the device, with dressing changes, and upon removal.

**Results:**

In 2018, there were a total of 14 CVC dislodgements and 12 in the year 2019. For the year 2020, there were 4 dislodgements and 2 in 2021. Both adult and pediatric patients utilized approximately 12 SASS. From the nurses who cared for the patients, 19 responses were obtained regarding the SASS. Overall, 94% of staff recommended this product for use. No accidental removal of the CVC was reported with use of the SASS. With cleaning around the catheter insertion site, 53% believed that they were able to clean better while 47% felt that it was the same with the previous practice. In terms of duration of changing the dressing and maintaining the catheter, 53% felt that it was the same while 47% thought it was faster. During maintenance, the most discomfort reported by patients was 4/10.

**Conclusions:**

The SASS can be implemented as another viable method to prevent accidental dislodgement of the CVC while securing CVC without sutures. In addition, RNs believed it allows for faster cleaning around the insertion site and faster dressing changes with minimal discomfort to the patient. More data can be collected over longer periods of time and to look at its efficacy in non-burn patients.

